# Survivin-responsive conditionally replicating adenovirus kills rhabdomyosarcoma stem cells more efficiently than their progeny

**DOI:** 10.1186/1479-5876-12-27

**Published:** 2014-01-27

**Authors:** Kiyonori Tanoue, Yuqing Wang, Minako Ikeda, Kaoru Mitsui, Rie Irie, Takao Setoguchi, Setsuro Komiya, Shoji Natsugoe, Ken-ichiro Kosai

**Affiliations:** 1 Department of Gene Therapy and Regenerative Medicine, Kagoshima University Graduate School of Medical and Dental Sciences, Kagoshima, Japan; 2 Department of Digestive Surgery, Breast and Thyroid Surgery, Kagoshima University Graduate School of Medical and Dental Sciences, Kagoshima, Japan; 3 The Near-Future Locomoter Organ Medicine Creation Course (Kusunoki Kai), Kagoshima University Graduate School of Medical and Dental Sciences, Kagoshima, Japan; 4 Department of Orthopaedic Surgery, Kagoshima University Graduate School of Medical and Dental Sciences, Kagoshima, Japan

**Keywords:** Cancer stem cells, Conditionally replicating adenovirus, Fibroblast growth factor receptor 3, Gene therapy, Oncolytic adenovirus, Promoter, Rhabdomyosarcoma, Survivin, Tumor-initiating cell, Virotherapy

## Abstract

**Background:**

Effective methods for eradicating cancer stem cells (CSCs), which are highly tumorigenic and resistant to conventional therapies, are urgently needed. Our previous studies demonstrated that survivin-responsive conditionally replicating adenoviruses regulated with multiple factors (Surv.m-CRAs), which selectively replicate in and kill a broad range of cancer-cell types, are promising anticancer agents. Here we examined the therapeutic potentials of a Surv.m-CRA against rhabdomyosarcoma stem cells (RSCs), in order to assess its clinical effectiveness and usefulness.

**Methods:**

Our previous study demonstrated that fibroblast growth factor receptor 3 (FGFR3) is a marker of RSCs. We examined survivin mRNA levels, survivin promoter activities, relative cytotoxicities of Surv.m-CRA in RSC-enriched (serum-minus) vs. RSC-exiguous (serum-plus) and FGFR3-positive vs. FGFR3-negative sorted rhabdomyosarcoma cells, and the *in vivo* therapeutic effects of Surv.m-CRAs on subcutaneous tumors in mice.

**Results:**

Both survivin mRNA levels and survivin promoter activities were significantly elevated under RSC-enriched relative to RSC-exiguous culture conditions, and the elevation was more prominent in FGFR3-positive vs. FGFR3-negative sorted cells than in RSC-enriched vs. RSC-exiguous conditions. Although Surv.m-CRA efficiently replicated and potently induced cell death in all populations of rhabdomyosarcoma cells, the cytotoxic effects were more pronounced in RSC-enriched or RSC-purified cells than in RSC-exiguous or progeny-purified cells. Injections of Surv.m-CRAs into tumor nodules generated by transplanting RSC-enriched cells induced significant death of rhabdomyosarcoma cells and regression of tumor nodules.

**Conclusions:**

The unique therapeutic features of Surv.m-CRA, *i.e*., not only its therapeutic effectiveness against all cell populations but also its increased effectiveness against CSCs, suggest that Surv.m-CRA is promising anticancer agent.

## Background

Accumulating data have suggested that cancer stem cells (CSCs), also called tumor-initiating cells, are a small but specialized population of tumor cells that possess high capacity for tumor initiation, invasion, and metastasis, as well as for self-renewal [1]. After most cells in the tumor are killed by conventional chemotherapy or radiotherapy, residual CSCs are believed to give rise to the bulk populations of tumor-cell progeny and recapitulate the original tumor nodule [2]. From the standpoints of clinical oncology and therapeutics, the most critical feature of CSCs is that they are highly resistant to conventional chemoradiotherapies [3,4], because they are predominantly in a dormant or slow-growing phase of the cell cycle [3] and they express high levels of multiple drug-resistance transporters [4]. Because the poor prognosis of patients with malignant tumors is caused, at least in part, by CSCs, the development of effective therapies against CSCs is urgently needed.

Rhabdomyosarcoma is the most common soft-tissue malignancy in children and adolescents [5]. Metastatic rhabdomyosarcoma is often incurable, and is associated with poor prognosis; approximately 20% of rhabdomyosarcoma patients have disseminated disease at the time of diagnosis [6]. Whereas current treatment for rhabdomyosarcoma relies on chemotherapy, the cytotoxic actions of chemotherapeutic agents are not only ineffective but also non–tumor-specific in treatment of advanced and metastatic tumors. Therefore, these agents can impair normal development and cause secondary cancers in some growing children [5]. To develop a novel and innovative therapy against malignant rhabdomyosarcoma, we previously identified rhabdomyosarcoma stem cells (RSCs) and showed that fibroblast growth factor receptor 3 (FGFR3) is a marker of RSCs [7]. For instance, implantation of a single FGFR3-positive KYM-1 rhabdomyosarcoma cell can form a tumor nodule *in vivo* consisting of histologically defined rhabdomyosarcoma cells, whereas a single FGFR3-negative cell cannot form such nodules [7]. Likewise, the careful analyses in our previous study characterized FGFR3-positive rhabdomyosarcoma cells as RSCs.

Conditionally replicating adenoviruses (CRAs), also called oncolytic adenoviruses, replicate predominantly in tumor cells, which they kill via apoptosis mediated by adenoviral proteins; therefore, CRAs are promising anticancer agents [8,9]. We previously developed a method to efficiently construct diverse CRAs that can specifically target and/or efficiently treat malignant tumors using multiple factors (m-CRAs) [10]. Our m-CRA construction system expedited the process of generating, modifying, and testing diverse m-CRAs with the goal of developing an ideal m-CRA for tumor therapy; indeed, our m-CRA strategy increased the potential cancer specificity of virotherapy [10-12]. Survivin, a new member of the inhibitor of apoptosis (IAP) gene family, is expressed at high levels in cancerous but not normal tissues, and high survivin expression levels are positively correlated with poor prognosis, an accelerated rate of recurrence, and increased resistance to therapy in cancer patients [13,14]. We developed several types of survivin-responsive m-CRAs (Surv.m-CRAs) in which adenoviral E1A was regulated by the promoter of survivin; in some versions of these viruses, the p53-binding domain in E1B was deleted (*i.e*., E1B55KD), the Rb-binding domain in E1A was deleted, or the native E1B promoter was replaced with another cancer-specific promoter [11,12]. All Surv.m-CRAs induced potent *in vitro* and *in vivo* cytotoxic effects against a variety of malignant tumors, and exhibited stronger and more cancer-selective phenotypes than telomerase reverse transcriptase (Tert)-responsive m-CRAs (Tert.m-CRAs), which are currently among the best CRAs [11,12]. Furthermore, certain types of Surv.m-CRAs significantly increased cancer specificity (*i.e*., safety) without reduced anticancer effects [11].

CSCs are resistant to conventional chemoradiotherapies, and the therapeutic potentials of Surv.m-CRAs against CSCs have not been well examined. In order to evaluate the clinical usefulness of Surv.m-CRAs against malignant and incurable tumors, it will be necessary to perform careful comparative studies of endogenous survivin expression levels, activity of transduced survivin promoters, and relative antitumor effects on CSCs and their progeny. More generally and importantly, it has not yet been clearly elucidated whether transcriptional targeting using CRAs is a useful strategy for treating CSCs. Because FGFR3-positive RSCs are a useful model for CSCs, we examined the biological features of survivin and compared the therapeutic potentials of Surv.m-CRA against RSCs and progeny tumor cells.

## Methods

### Cells and cell culture

KYM-1 cell lines were purchased from Health Sciences Research Resources Bank (Tokyo, Japan). KYM-1 cells were cultured in DMEM, supplemented with 10% FCS, 100 units/ml penicillin G, and 100 μg/ml streptomycin (Invitrogen, Carlsbad, CA, USA). In some experiments, KYM-1 cells were cultured in serum-free S-Clone (Eidia Co., Ltd, Tokyo, Japan) containing 10 ng/ml basic fibroblast growth factor (bFGF).

### Flow-cytometric analysis and cell sorting

Cells were conjugated with anti-FGFR3 antibody (R & D, Minneapolis, MN, USA) for 30 min on ice. Cells were resuspended in the same buffer at 1.0 × 10^7^ per ml, and then kept on ice until analysis. Flow-cytometric analysis was performed using CyAn™ ADP (Beckman Coulter, Fullerton, CA, USA). For further analyses, cells were sorted using a FACSAriaII (BD Biosciences, San Jose, CA) to isolate pure populations of FGFR3-positive and FGFR3-negative cells.

### Generation of adenoviruses

The following viruses were propagated and purified as described previously [15-19]: E1-deleted replication-defective adenoviruses; two types of Ads-LacZ that expressed the LacZ gene under the transcriptional control of the Rous sarcoma virus long terminal repeat (RSV promoter) (Ad.RSV-LacZ) or the survivin promoter (Ad.Surv-LacZ); two types of Ads-EGFP that expressed the enhanced green fluorescent protein (EGFP) gene under the cytomegalovirus immediate early gene enhancer/promoter (CMV promoter) (Ad.CMV-EGFP) or the cytomegalovirus enhancer and β-actin promoter (CA promoter) (Ad.CA-EGFP); Ad.dE1.3 that expressed no gene, and Ad.CA-EGFP/RGD, in which an Arg-Gly-Asp (RGD)-containing peptide was added to the HI loop of the fiber-knob domain of Ad.CA-EGFP. Surv.m-CRA with wild-type E1A downstream of the survivin promoter, E1B55KD downstream of the CMV promoter, and the EGFP gene downstream of the CMV promoter was generated as described previously and used for this study [10-12].

### Adenoviral gene transduction efficiencies

The adenoviral gene transduction efficiency (AGTE) for each cell type *in vitro* was assessed by infecting cells with Ad.CMV-EGFP at several different multiplicities of infection (MOIs), detaching the cells 48 h after infection, and analyzing the percentage of EGFP-positive cells by flow cytometry [20].

### Promoter activities

Promoter activities were examined as described previously with some modification [15,21]. Briefly, cells (8 × 10^5^ cells per plate) were infected with Ad.Surv-LacZ or Ad.RSV-LacZ at an MOI of 30 for 1 h, and then incubated with fresh media. The cells were collected 48 h post-infection, and β-gal activity was measured using the β-Galactosidase Enzyme Assay System (Promega, Madison, WI, USA) as described previously [15,21]. In addition, expression levels of β-galactosidase in individual KYM-1 cells were examined by flow cytometry using the FluoReporter lacZ Flow Cytometry Kit (Molecular Probes, Leiden, The Netherlands).

### Real time quantitative reverse transcription–polymerase chain reaction (qRT-PCR) analysis

RNA was isolated using Sepasol-RNA I Super G (Nacalai Tesque, Kyoto, Japan) or the CellAmp Direct RNA Prep Kit (Takara Bio Inc., Ootsu, Japan), and was subsequently reverse-transcribed using the PrimeScript II First Strand cDNA Synthesis Kit (Takara Bio Inc.) [22,23]. RT-PCR using QuantiFast SYBR Green PCR (Qiagen, Venlo, The Netherlands) was performed on a Rotor Gene RG-3000 (Qiagen). The relative mRNA expression levels were determined by the comparative C_t_ method; expression levels of individual genes were normalized against the levels of the reference gene *HPRT*, which encodes hypoxanthine guanine phosphoribosyl transferase. The following primer sets and annealing temperatures were used: survivin, 5- CCAGTGTTTCTTCTGCTTCAA-3 and 5-GAATGCTTTTTATGTTCCTCTATG-3 at 60°C; *HPRT*, 5- TGACCTTGATTTATTTTGCATACC-3 and 5-CTCGAGCAAGACGTTCAGTC-3 at 60°C [11,12].

### Cytotoxic effects *in vitro*

Cells in 96-well plates were infected with each adenovirus at an MOI of 1, and cell viability was determined after 3 and 5 days using the WST-8 assay (Dojindo Laboratories, Mashiki, Japan) as described previously [11,12,24].

### Therapeutic effects *in vivo* in animal experiments

KYM-1 cells (1 × 10^6^ cells), which had been cultured in serum-minus media containing S-Clone and 10 ng/ml bFGF, were mixed with Matrigel (BD Biosciences) and subcutaneously inoculated into 5-week-old BALB/c nude mice. After a tumor nodule reached 6–10 mm in diameter, the mice were randomly divided into three groups. On day 0, a mouse in each group was given a single intratumoral injection of 150 μL of buffer (10 mmol/L Tris–HCl pH 7.4, 1 mmol/L MgCl_2_, 10% glycerol, and 20 μg/mL hexadimethrine bromide) containing 1 × 10^9^ plaque-forming units (pfu) of Surv.m-CRA (n = 6), Ad.dE1.3 (n = 7), or phosphate-buffered saline (PBS) (n = 8). Subsequently, tumor size was measured twice a week, and tumor volume was calculated according to the following formula: volume = long axis × (short axis)^2^ × 0.5.

For histopathologic analysis, tumors were fixed in 10% buffered formalin, embedded in paraffin, cut into 4-μm sections, and stained with hematoxylin and eosin.

All animal studies were performed in accordance with National Institutes of Health guidelines and with the approval of the Division of Laboratory Animal Science, Natural Science Center for Research and Education, Kagoshima University. All reasonable efforts were made to minimize suffering.

### Statistical analysis

Data were represented as the means ± standard errors (s.e.). Statistical significance was determined using Student’s *t* test. *P* < 0.05 was defined as statistically significant.

## Results

### RSCs can be maintained, expanded, and differentiated *in vitro*

**Figure 1:**
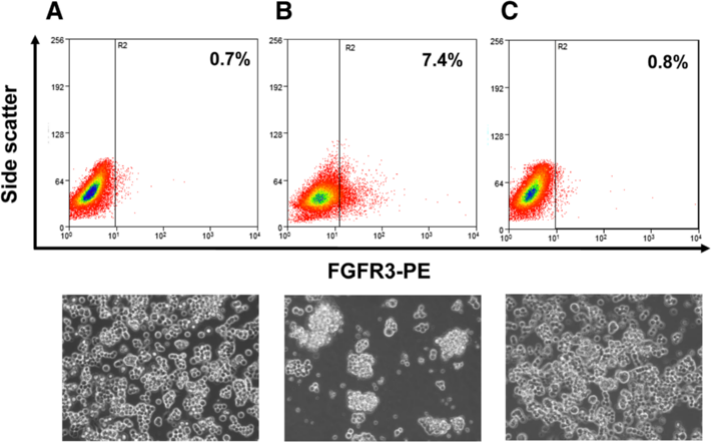
**The percentage of FGFR3**-**positive cells determined by flow**-**cytometric analysis (upper), and microscopic image of KYM-1 cells (200× magnification; lower) in each culture condition.** KYM-1 cells were cultured in serum-plus media **(A)**, then cultured for 3 weeks in serum-minus media containing 10 ng/ml bFGF **(B)**, and subsequently cultured in the serum-plus media for 1 week **(C)**. The percentages of FGFR3-positive cells and spheres were significantly increased by the serum-minus culture and decreased by serum-plus culture.

We characterized FGFR3-positive KYM-1 cells as RSCs in a previous study [7]. Therefore, in this study, we first reproduced two distinct culture conditions that successfully induced FGFR3-positive RSC-enriched and FGFR3-negative RSC-exiguous cell fractions *in vitro*. Flow-cytometric analysis revealed that FGFR3-positive cells accounted for only 0.7% of the population when cells were cultured in regular serum-plus media (Figure 1A). However, the percentage of FGFR3-positive cells significantly increased, to 7.4% of the population, after 3-week culture in serum-minus media (Figure 1B). The population of FGFR3-positive cells was drastically decreased to 0.8%, *i.e*., almost to the original level, only 1 week after the RSC-enriched cell fractions were returned to culture in regular serum-plus media (Figure 1C). Sphere formation, which represented clonogenic growth of the floating cells and was therefore a useful indication of enrichment of CSCs, was observed solely in the serum-minus condition. Thus, we established two distinct *in vitro* conditions that allow accurate assessments of survivin and Surv.m-CRAs in RSCs and their progeny.

### AGTEs in KYM-1 cells

**Figure 2:**
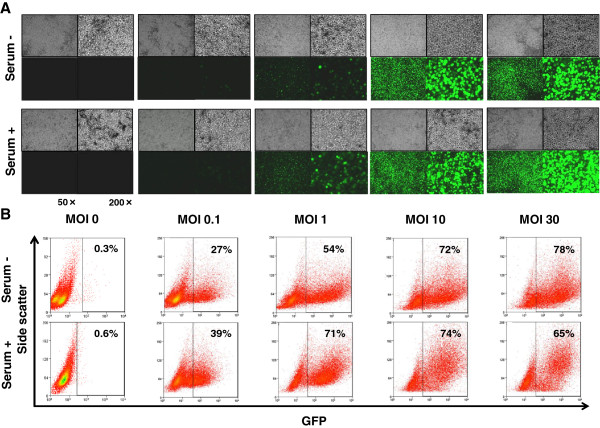
**Adenoviral gene transduction efficiencies (AGTEs) in each culture condition.** Cells that had been cultured under either serum-minus or serum-plus conditions were infected for 48 h with Ad.CMV-EGFP at MOI of 0 (none), 0.1, 1, 10, or 30. Subsequently, fluorescence-microscopic analysis **(A)** and flow-cytometric analysis **(B)** were performed to assess EGFP-expressing cells. **(A)** Representative images of phase-contrast (upper) and the fluorescence (lower) microscopy are shown at 50× (left) and 200× (right) magnification. No difference in AGTEs between the two groups was apparent.

**Figure 3:**
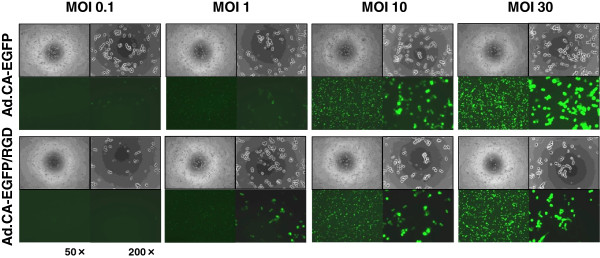
**AGTEs of adenoviral vectors with wild or modified fiber.** KYM-1 cells were infected with either Ad.CA-EGFP or Ad.CA-EGFP/RGD, which have wild-type or modified fiber (RGD-peptide added to the fiber knob), respectively, at an MOI of 0.1, 1, 10, or 30, and then observed under phase-contrast and fluorescence microscopy 48 h later. Representative phase-contrast (upper) and fluorescence (lower) images are shown at 50× (left) and 200× (right) magnification. There was no apparent difference in AGTEs between the two groups.

KYM-1 cells, cultured in either serum-minus or serum-plus media, were infected with Ad.CMV-EGFP at various MOIs and analyzed by fluorescence microscopy or flow cytometry to assess AGTEs (Figure 2). AGTEs in KYM-1 cells were higher than those in other previously examined cell types [11,12]; however, the difference in AGTEs between RSC-enriched and RSC-exiguous cells was not significant, or at least was not drastic. We further compared AGTEs between infections with Ad.CA-EGFP and Ad.CA-EGFP/RGD, because a number of previous studies demonstrated that modification of the fiber knob with the RGD peptide increased AGTEs in some cell types [19]. Fiber modification did not drastically increase or change AGTEs in KYM-1 cells, either in RSC-enriched or RSC-exiguous fractions (Figure 3). Based on these results, we decided to use replication-defective adenoviral vectors and m-CRAs with wild-type fibers for subsequent experiments.

### Higher expression levels of *survivin* mRNA in the FGFR3-positive KYM-1 cell

**Figure 4:**
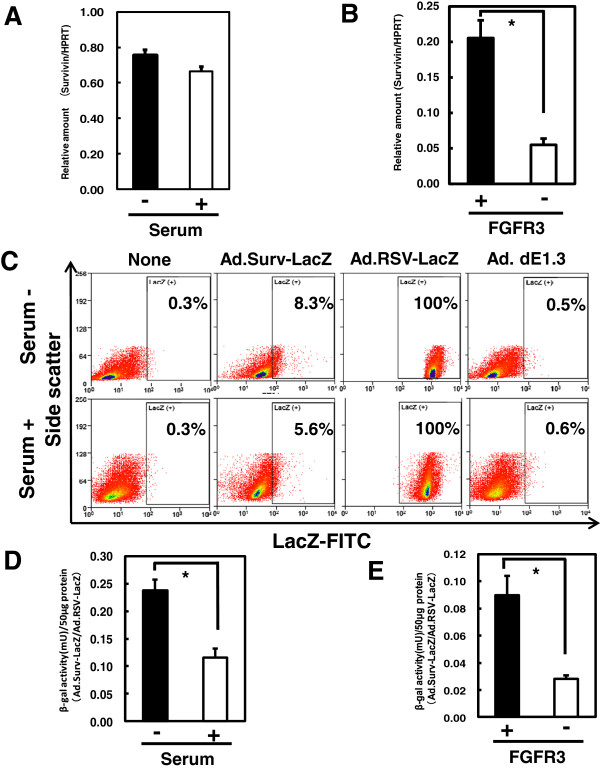
**Survivin mRNA expressions and survivin promoter activity. (A, B)** Expression levels of survivin mRNA under serum-minus and serum-plus conditions **(A)** and in sorted FGFR3-positive and FGFR3-negative cells **(B)** were measured by real-time qRT-PCR. The *HPRT* gene was amplified as an internal control. **(C)** Flow-cytometric analysis to detect LacZ-expressing cells 48 h after Ad.Surv-LacZ or Ad.RSV-LacZ infection at MOI of 30 demonstrated the activities of the survivin and control RSV promoters in individual cells. **(D, E)** β-galactosidase activities in cells cultured in serum-minus or serum-plus media **(D)** and in sorted FGFR3-positive and FGFR3-negative cells **(E)** were measured after the same infection described in **(C)**. *, *P* < 0.05.

To assess whether the expression levels of survivin mRNA were changed in RSCs, we performed qRT-PCR analyses to make comparisons between RSC-enriched (serum-minus) and RSC-exiguous (serum-plus) cell fractions and between sorted FGFR3-positive and FGFR3-negative cells. The RSC-enriched cell fraction exhibited a slight increase in survivin mRNA level; however, the difference between the RSC-enriched and RSC-exiguous cell fractions was not statistically significant (Figure 4A). We next examined survivin mRNA levels in sorted FGFR3-postive and FGFR3-negative cells, because such an examination in purified FGFR3-positive cells should make their characteristic phenotypes more apparent. In accordance with that speculation, survivin mRNA levels in sorted FGFR3-positive KYM-1 cells were significantly higher than in FGFR3-negative cells (Figure 4B). These results suggest that survivin is more highly expressed in FGFR3-positive RSCs than in FGFR3-negative progeny.

### Higher activities of survivin promoter in FGFR3-positive RSCs

We examined the activities of the survivin and control RSV promoters using replication-defective adenoviral vectors with the LacZ gene downstream of each of these promoters. Flow-cytometric analysis revealed that almost 100% of cells expressed LacZ after Ad.RSV-LacZ infection, and that less than 0.6% of cells were nonspecifically positive after no infection or infection with Ad.dE1.3 (lacking the transgene); thus, the experimental conditions were appropriate (Figure 4C). The percentages of cells expressing LacZ under control of the survivin promoter were 8.3 and 5.6% under the RSC-enriched (serum-minus) and RSC-exiguous (serum-plus) conditions, respectively. Next, we examined the activity of the survivin promoter relative to that of the RSV promoter, a representative control promoter with constitutively strong activity, by measuring β-galactosidase activities after infection with Ad.Surv-LacZ (Figure 4D and E). The survivin promoter activity was significantly higher in RSC-enriched (serum-minus) than in RSC-exiguous (serum-plus) conditions, and also significantly higher in sorted FGFR3-positive than in FGFR3-negative cells. This result, together with the same tendency in endogenous survivin gene expression, suggests that the survivin promoter, which is highly active in tumor cells but minimally active in normal cells [11,12], is more active in RSCs than in progeny. Furthermore, these results confirm that the adenovirally transduced promoter region functions well in RSCs.

### Surv.m-CRA exhibited more efficient replication and cytotoxicity against RSC-enriched cell fractions *in vitro*

**Figure 5:**
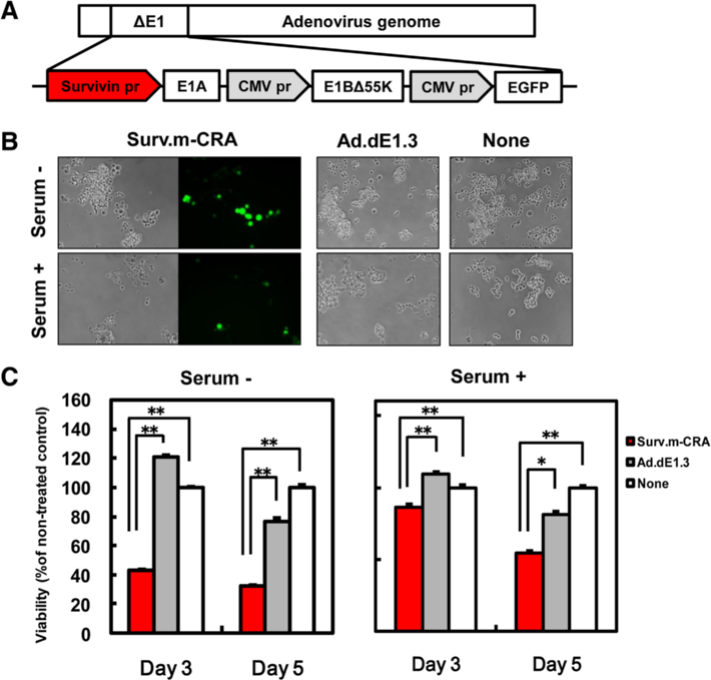
**Viral replication of Surv.m-CRA and cytotoxicity against KYM-1 cells cultured in serum-minus and serum-plus media. (A)** Schematic representation of the construction of Surv.m-CRA. **(B)** Representative images of phase-contrast and fluorescence-microscopic pictures 5 days after infection with Surv.m-CRA, Ad.dE1.3, or no virus (none) of KYM-1 cells cultured in serum-minus or serum-plus media. Viral replication and cytopathic effects were prominent in both groups after Surv.m-CRA infection. **(C)** Cell viability was determined by WST-8 assay 3 or 5 days after infection.*, *P* < 0.05. **, *P* < 0.005. Surv.m-CRA efficiently replicated and potently induced cell death in both cell fractions cultured in serum-minus and serum-plus media.

The construction of the Surv.m-CRA used in this study is shown in Figure 5A. We first explored apparent viral replication and the cytotoxic effects of Surv.m-CRAs against RSC-enriched and RSC-exiguous cell fractions by microscopically observing the spread of cells expressing EGFP and the swollen cells that are a characteristic feature of the adenoviral cytopathic effect (Figure 5B). In both fractions, Surv.m-CRA induced prominent viral replication and cytotoxic effects as early as 3 days after infection at an MOI of 1. To accurately and quantitatively analyze the cytotoxic effect, we performed the WST viability assay (Figure 5C). Surv.m-CRA potently induced cell death in both groups. The cytotoxicity was somewhat more prominent under the RSC-enriched (serum-minus) than the RSC-exiguous (serum-plus) conditions (e.g., *P* < 0.005 and *P* < 0.05 between Surv.m-CRA and Ad.dE1.3 in RSC-enriched and RSC-exiguous, respectively, on day 5).

### Surv.m-CRA exhibited more efficient replication and cytotoxicity against sorted FGFR3-positive cells *in vitro*

**Figure 6:**
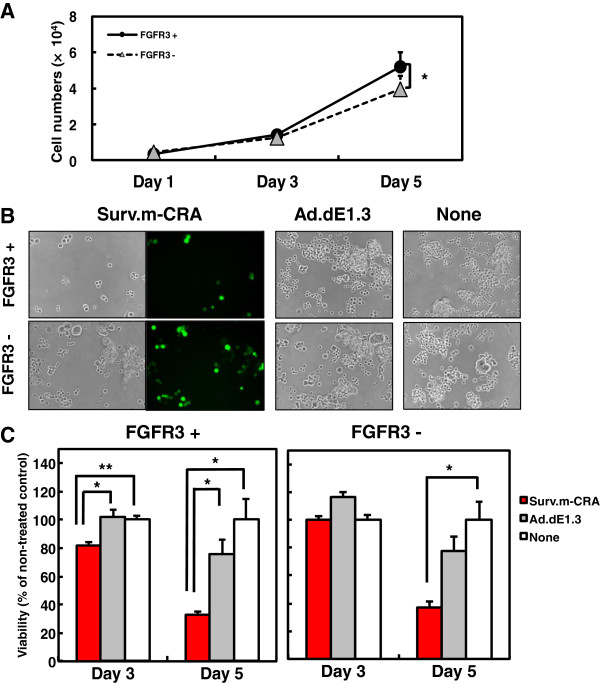
**Viral replication of Surv.m**-**CRA and cytotoxicity against sorted FGFR3**-**positive and FGFR3-negative cells. (A)** Growth curves of sorted cells. Whereas FGFR3-positive cells proliferated somewhat more rapidly than FGFR3-negative cells, the difference was not drastic. *, *P* < 0.05. **(B)** Representative images of phase-contrast and the fluorescence-microscopic pictures 5 days after infection with Surv.m-CRA, Ad.dE1.3, or no virus. Whereas viral replication and cytopathic effects of Surv.m-CRA were prominent in both groups, the proportion of remaining cells 5 days after Surv.m-CRA infection at an MOI of 1 was lower in sorted FGFR3-positive cells than in sorted FGFR3-negative cells. **(C)** Cell viability was determined by WST-8 assay 3 or 5 days after infection. Cytotoxic effects of Surv.m-CRA were statistically significantly higher than those of Ad.dE1.3 solely in the sorted FGFR3-positive cells. *, *P* < 0.05; **, *P* < 0.005 between Surv.m-CRA and either Ad.dE1.3 or no virus.

**Figure 7:**
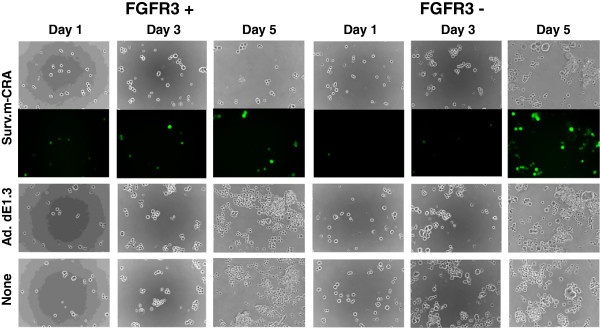
**Viral replication of Surv.m-CRA and cytotoxicity against sorted FGFR3-positive and FGFR3-negative cells.** Sorted FGFR3-positive and FGFR3-negative cells were infected with Surv.m-CRA, Ad.dE1.3, or no virus (none) at an MOI of 1. The cells were observed under phase-contrast and fluorescence microscopy 1, 3, and 5 days after infection. Representative phase-contrast (upper) and fluorescence (lower) images are shown at 50× magnification. In the case of no infection (none), FGFR3-positive cells proliferated more rapidly than FGFR3-negative cells after 3 and 5 days. By contrast, the proportion of remaining cells 5 days after Surv.m-CRA infection was lower in FGFR3-positive than in FGFR3-negative cells. These results suggest that Surv.m-CRA exhibited more efficient replication in and cytotoxicity against sorted FGFR3-positive than FGFR3-negative cells.

To directly examine the therapeutic potentials of Surv.m-CRA against RSCs and progeny, FGFR3-positive and -negative cells were sorted and subsequently cultured in serum-minus and serum-plus media, respectively. Whereas FGFR3-positive cells proliferated somewhat more rapidly than FGFR3-negative cells after sorting and regrowth, the difference was not drastic (Figure 6A and Figure 7). Both cell types were infected with Surv.m-CRA, the control Ad.dE1.3, or no virus (none) 3 days after the sorting (*i.e*., when the cells had recovered from possible damage related to the sorting procedure), and viral replication and cytotoxic effects were assessed 1, 3, or 5 days later. Efficient viral replication, assessed by the spread of EGFP-expressing cells, was observed in both cell types (Figure 6B). In the case of cells infected with Surv.m-CRA, the number of FGFR3-positive cells was noticeably smaller than the number of FGFR3-negative cells after 5 days of growth (Figure 6B and Figure 7). This result suggests that viral replication and the resulting cytotoxic effects of Surv.m-CRA were more prominent in RSCs than in the progeny. According to the microscopic analysis, the viability assay accurately and quantitatively demonstrated that the cytotoxicity of Surv.m-CRA was higher in FGFR3-positive RSCs than in FGFR3-negative progeny (Figure 6C and Figure 7). A statistically significant difference in the percentage of viable cells between Surv.m-CRA–treated and the control replication-defective Ad.dE1.3–treated groups was observed in sorted FGFR3-positive cells, but not in sorted FGFR3-negative cells. Together, the results from all the *in vitro* experiments revealed that Surv.m-CRA induced prominent viral replication and cell death in all rhabdomyosarcoma cell fractions, including RSCs, and that such effects were more potent in RSCs than in progeny. This is consistent with the higher survivin expression levels and promoter activities in RSCs relative to progeny, shown above (Figure 3).

### Surv.m-CRA had potent *in vivo* therapeutic effects on tumors generated from RSC-enriched rhabdomyosarcoma cells in mice

**Figure 8:**
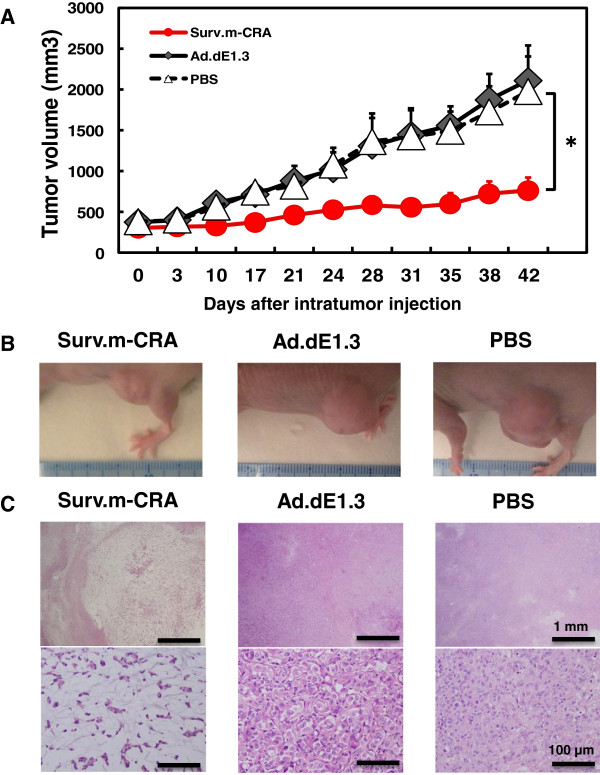
***In vivo *therapeutic effects of Surv.m-CRA against tumors in mice.** Tumor nodules were generated in mice by implantation of RSC-enriched rhabdomyosarcoma cells, and a single intratumoral injection of Surv.m-CRA, Ad.dE1.3, or PBS was subsequently administered to each nodule. **(A)** The macroscopic tumor size after each treatment. A significant difference was found between mice treated with Surv.m-CRA and those treated with either control (Ad.dE1.3 or PBS) (*P* < 0.05). **(B)** Representative macroscopic pictures of a tumor nodule 42 days after each treatment. **(C)** Representative histopathologic pictures of hematoxylin/eosin-stained sections in the tumor nodule 42 days after each treatment. In Ad.dE1.3-treated and the PBS-treated mice, tumor nodules mainly consisted of viable tumor cells exhibiting malignant features without large necrotic areas. By contrast, in Surv.m-CRA–treated mice, tumor nodules mainly consisted of large necrotic areas with loose connective tissues and without viable tumor cells. Original magnification: 40× (top; scale bar, 1 mm) and 400× (bottom; scale bar, 100 mm).

We subcutaneously inoculated RSC-enriched rhabdomyosarcoma cells into mice to generate a tumor nodule, into which Surv.m-CRA (1 × 10^9^ pfu), the control Ad.dE1.3, or no virus (none) was subsequently directly injected. Periodic measurement of tumor size revealed that a single intratumoral injection of Surv.m-CRA significantly inhibited tumor growth in comparison to Ad.dE1.3 42 days after adenoviral injection (Figure 8A, B). By contrast, there was no significant difference in tumor size on day 42 between the controls, *i.e*., Ad.dE1.3–treated and PBS–treated mice. Furthermore, histopathologic analysis of viable and dead tumor cells clearly demonstrated the potent therapeutic effects of Surv.m-CRAs beyond what could be shown by the macroscopic analysis (Figure 8B, C): the macroscopically large tumor nodules in control mice treated with either Ad.dE1.3 or PBS consisted mainly of viable tumor cells with histological features of active malignancy, including a large number of mitoses, heterogeneous morphologies of nuclei and cells, and densely accumulated cells; in addition, these tumors contained small and spotty areas consisting of spontaneous necroses in their centers (Figure 8B, C). By contrast, the macroscopically small nodules in the Surv.m-CRA–treated mice consisted histologically of large necrotic areas with loose connective tissues but no apparent viable malignant cells. Thus, a single injection of Surv.m-CRA into tumor nodules generated by implantation of RSC-enriched rhabdomyosarcoma cells in mice exhibited a potent therapeutic effect *in vivo*.

## Discussion

We previously showed that Surv.m-CRAs could treat a broad range of cancer types more efficiently and safely (*i.e*., cancer-specifically) than Tert.m-CRAs, which are among the best CRAs [11,12,25]. The results of this study demonstrate not only that Surv.m-CRAs can efficiently kill all populations of rhabdomyosarcoma cells, including both RSCs and their progeny (*i.e*., the bulk of malignant cells), but also that the antitumor effects of Surv.m-CRAs are higher against RSCs. This feature is clinically meaningful and promising because the therapeutic mode of Surv.m-CRAs is opposite to that of conventional chemoradiotherapies, and because Surv.m-CRA may overcome, at least in part, the serious drawbacks of current cancer treatments.

Some previous studies assessed the cytotoxic effects of oncolytic viruses on CSCs [26], and several groups reported that their CRAs might be effective against stem-like cancer cells of glioblastoma [27,28], esophageal cancer [29], and breast cancer [30]. However, the efficacy of these CRAs against CSCs was not accurately established in these studies, due to limitations of the analyses or the CSC models used. From the standpoint of the analyses, the lack of point-by-point comparisons of biological features, both regarding genes that regulated viral replication and the differences in the effects of CRAs between sorted CSCs and progeny, led to unclear conclusions regarding the most important question: how efficiently do these viruses kill CSCs in comparison to their progeny [27,28]? From the standpoint of the CSC models, some previous studies used radioresistant cancer cells as cancer stem-like cells, but did not examine sorted CSCs [28]. Although radiation treatment may enrich CSCs, the radioresistant cancer cells are not equivalent to CSCs. Thus, although the previous studies did provide some important information, their results may not allow a generalized assessment regarding the potentials of CRAs against CSCs. Therefore, the efficacy of each oncolytic virus and CRA against CSCs should be individually and carefully assessed in the proper experimental models.

To accurately assess the biological features and therapeutic potential of Surv.m-CRAs against CSCs, in a previous study we identified FGFR3 as a useful marker that allows accurate monitoring and purification of RSCs; a single implanted FGFR3-positive rhabdomyosarcoma cell could form a tumor *in vivo*, whereas FGFR3-negative cells did not form tumors [7]. Because FGFR3-positive rhabdomyosarcoma cells, including KYM-1 cells, were characterized as RSCs in our previous study, it was not necessary to repeat this characterization in this study. Based on those results, in this study we carefully compared the biological features of survivin (both endogenous gene expression and the transduced promoter activity) and Surv.m-CRAs, both between RSC-enriched and RSC-exiguous conditions and between purified FGFR3-positive RSCs and purified FGFR3-negative progeny cells. We used both of these experimental systems because the former (enriched CSCs together with some progeny cells) may, at least in part, reflect the *in vivo* microenvironment, whereas the latter (purified CSCs) may facilitate clarification of the biological differences between CSCs and progenitor cells. Analyses in both experimental systems clearly revealed that the activity of the survivin promoter, which has already been shown to have stronger and more cancer-specific activity than the Tert promoter [11,12], was further increased in RSCs; indeed, Surv.m-CRAs efficiently killed all populations with the desirable property of increased therapeutic efficacy against RSCs.

On the other hand, the movements and changes of CSCs within the body are not fully understood, and these points can be accurately addressed in only a few animal models. In addition, human type 5 adenovirus, which is the backbone of Surv.m-CRAs, can infect mouse cells but cannot replicate in mice; therefore, there is no available animal model in which the therapeutic efficacy of CRAs against CSCs can be accurately analyzed. Therefore, we assessed the therapeutic efficiency of Surv.m-CRAs in tumor nodules generated by implantation of RSC-enriched rhabdomyosarcoma cells; Surv.m-CRAs exhibited a potent *in vivo* therapeutic effect in this animal model. Although the *in vivo* efficacy of Surv.m-CRAs against CSCs cannot be quantitatively assessed, this result demonstrates the therapeutic efficacy and the possible clinical utility of Surv.m-CRAs for treating rhabdomyosarcoma.

The RGD-based fiber modification allows the adenovirus to use integrins as alternative receptors during the cell entry process, and increases AGTEs in certain cell types, particularly those that lack the expression of the native Coxsackie-adenovirus receptor [31]. In contrast to a previous report that fiber-modified CRA increased therapeutic efficacy against CSCs of glioma [27], in our hands the fiber modification did not drastically increase AGTEs. Therefore, we did not need to modify the fibers of Surv.m-CRAs in order to obtain therapeutic benefits, at least in this model. The clinical utility of the fiber modification may depend on the adenoviral infectivity in each cell type.

Together with the previous findings, the results of this study suggest the possible therapeutic efficacy of Surv.m-CRAs against other types of CSCs. Clinical studies previously demonstrated that high survivin expression levels are positively correlated with poor prognosis, accelerated rate of recurrence, and increased resistance to therapy in a variety of cancer types, including rhabdomyosarcoma [5,13,14]. Our results reported here regarding up-regulated survivin expression and survivin promoter activity in RSCs are consistent with the clinical findings, and should therefore be considered reasonable. Because a close relationship between higher expression levels of survivin and more malignant phenotypes has been observed in a variety of cancer types, the potent efficacy of Surv.m-CRAs to the RSCs revealed in this study may be applicable to other types of CSCs.

In terms of mechanism, accumulated data have revealed that survivin is involved in apoptosis resistance and proliferation of cancer cells, mediated at least in part through the responses to various growth factors, including bFGF [32,33]. bFGF up-regulates survivin expression in certain cancer cells [32], and survivin plays an essential role in angiogenesis in tumors by up-regulating bFGF expression [33], leading to activation of the FGFR3-mediated signaling pathway [7]. Any mechanistic inference based on these findings would necessarily be speculative, however, and the overall molecular mechanism underlying the relationship between the survivin expression and malignant features of CSCs should be clarified by extensive future studies.

**Figure 9:**
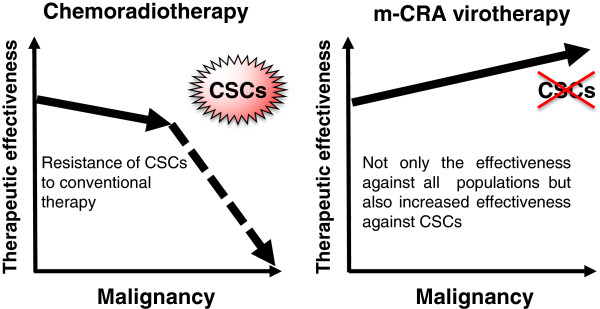
**The putative therapeutic advantage of m-CRA virotherapy.** In general, the therapeutic effectiveness of chemoradiotherapy is decreased in the advanced malignant tumor cells, and in particular, CSCs are resistant to conventional chemoradiotherapies. In contrast, not only m-CRA virotherapy can efficiently kill all populations of malignant tumor cells, including CSCs and their progeny, but also the antitumor effects of m-CRA virotherapy are higher against CSCs.

This study provides general and important information that should be useful in the development of oncolytic virotherapies against CSCs. To date, there have been very few successful reports of transcriptional targeting of oncolytic viruses against CSCs. In particular, none of the previous reports clearly showed that oncolytic viruses successfully acquired increased therapeutic efficacy against CSCs in parallel to increases in promoter activity and expression of a target gene. In this study, expression and promoter activity of survivin were further increased in CSCs; as a result of these transcriptional changes, Surv.m-CRAs exerted increased therapeutic efficacy against CSCs. Although the replicative mechanisms of adenoviruses have not been fully elucidated, the results described here suggest that the promising features of Surv.m-CRA may be due partly to specific features of adenoviruses and partly to the function of the survivin gene. Taken together, these findings demonstrate that Surv.m-CRA is an effective anticancer agent, but more generally, the results indicate that the use of m-CRAs represents a promising strategy for the development of effective anticancer agents against CSCs (Figure 9). In other words, the results described here pave the way to future development of several effective m-CRA–based therapies against CSCs; future progress will proceed via identification of new genes that target CSCs and generation of new m-CRAs using the promoters of these genes.

## Conclusion

Surv.m-CRAs demonstrated not only therapeutic efficacy against all the populations of rhabdomyosarcoma, but also increased efficacy against RSCs. These results will facilitate the clinical application of Surv.m-CRAs, and should be useful for future development of oncolytic virotherapies that target CSCs.

## Abbreviations

CSCs: Cancer stem cells; Surv.m-CRAs: Survivin-responsive conditionally replicating adenoviruses regulated with multiple factors; RSCs: Rhabdomyosarcoma stem cells; FGFR3: Fibroblast growth factor receptor 3; CRAs: Conditionally replicating adenoviruses; AGTE: The adenoviral gene transduction efficiency; IAP: Inhibitor of apoptosis; Tert.m-CRAs: Telomerase reverse transcriptase-responsive m-CRAs; bFGF: basic fibroblast growth factor; RSV promoter: Rous sarcoma virus long terminal repeat; EGFP: Enhanced green fluorescent protein; CMV: Cytomegalovirus; MOI: Multiplicities of infection; PBS: Phosphate-buffered saline.

## Competing interests

K. Kosai is the founder of WyK BiotechPharma Inc., but does not earn a salary from the company. All other authors declare no competing interest.

## Authors’ contributions

KT and YW designed the experimental protocol, performed the most of experiments, analyzed the data, and wrote the manuscript. MI, KM, and RI performed some experiments. TS provided the materials and information regarding rhabdomyosarcoma stem cells. SK and SN partially supervised the preclinical study. KK conceived and designed the study, supervised all the experiments, assessed the data, and wrote the manuscript. All authors read and approved the final manuscript.
